# Determinants of early child development in rural Tanzania

**DOI:** 10.1186/s13034-018-0224-5

**Published:** 2018-03-20

**Authors:** Ingeborg G. Ribe, Erling Svensen, Britt A. Lyngmo, Estomih Mduma, Sven G. Hinderaker

**Affiliations:** 1 University of Bergen, Bergen, Norway; 2 Haydom Lutheran Hospital, Haydom, Mbulu District, Tanzania

**Keywords:** Child development, Mental development, Cognitive development, Bayley Scales of Infant, Toddler development

## Abstract

**Background:**

It has been estimated that more than 200 million children under the age of five do not reach their full potential in cognitive development. Much of what we know about brain development is based on research from high-income countries. There is limited evidence on the determinants of early child development in low-income countries, especially rural sub-Saharan Africa. The present study aimed to identify the determinants of cognitive development in children living in villages surrounding Haydom, a rural area in north-central Tanzania.

**Methods:**

This cohort study is part of the MAL-ED (The Interactions of Malnutrition & Enteric Infections: Consequences for Child Health and Development) multi-country consortium studying risk factors for ill health and poor development in children. Descriptive analysis and linear regression analyses were performed. Associations between nutritional status, socio-economic status, and home environment at 6 months of age and cognitive outcomes at 15 months of age were studied. The third edition of the Bayley Scales for Infant and Toddler Development was used to assess cognitive, language and motor development.

**Results:**

There were 262 children enrolled into the study, and this present analysis included the 137 children with data for 15-month Bayley scores. Univariate regression analysis, weight-for-age and weight-for-length z-scores at 6 months were significantly associated with 15-month Bayley gross motor score, but not with other 15-month Bayley scores. Length-for-age z-scores at 6 months were not significantly associated with 15-month Bayley scores. The socio-economic status, measured by a set of assets and monthly income was significantly associated with 15-month Bayley cognitive score, but not with language, motor, nor total 15-month Bayley scores. Other socio-economic variables were not significantly associated with 15-month Bayley scores. No significant associations were found between the home environment and 15-month Bayley scores. In multivariate regression analyses we found higher Bayley scores for girls and higher Bayley scores in families with more assets. Adjusted R-squared of this model was 8%.

**Conclusion:**

We conclude that poverty is associated with a slower cognitive development in children and malnutrition is associated with slower gross motor development. This information should encourage authorities and other stakeholders to invest in improved welfare and nutrition programmes for children from early infancy.

## Background

It has been estimated that more than 200 million children under the age of five do not reach their full potential in cognitive development. This phenomenon is largely due to mechanisms that can be influenced; such as poverty, poor nutrition, and suboptimal care in the home [1]. We know that intervention programmes in the early years could prevent delay in development [2]. A life-course perspective shows that early child development affects future educational and occupational opportunities, and it may also determine a person’s risk of physical health in terms of obesity, malnutrition and mental-health problems [3]. Failure to thrive cognitively not only adversely affects the individual, but collectively limits national development. The cycle continues as it may be passed on to future generations and the gap of health inequities grows.

The first years of life constitute a critical period in brain development and functions [4]. Certainly genetic disposition plays a role [5], but external environmental factors are also important, including [3] nutritional status, socio-economic status, and home environment. Early interventions that influence these external factors may be effective in assuring children a good start [6], and may not only benefit the individual but also the society as a whole [7].

Much of what we know about early child development is based on research from high-income countries [8]. One study from rural Kenya analyzed the prevalence and risk factors of neurological disability and impairment in 6–9 year-olds. They found that moderate to severe cognitive impairment was present in 3% of children, and neonatal insult was the only risk factor identified [9]. However, to our knowledge, there is limited evidence on the determinants of early child development of the general population in low-income countries, and more specifically from rural sub-Saharan Africa.

A multicenter cohort study called MAL-ED (The Interactions of Malnutrition and Enteric Infections: Consequences for Child Health and Development) was started with one of the field sites in rural Tanzania. The study includes many items related to normal child development and aims to identify determinants of early child development in children. Specifically, the objectives of this paper were to find the associations between nutritional status, socio-economic status, and home environment—all at 6 months of age—and cognitive outcomes at 15 months of age in children living in villages surrounding Haydom, a rural area in north-central Tanzania.

## Methods

This study is part of the MAL-ED (The Interactions of Malnutrition & Enteric Infections: Consequences for Child Health and Development) [10] multi-country consortium studying risk factors for ill health and poor development in children. In this paper, data from the Tanzanian site (TZH) was analyzed.

### Study design

The study had a prospective cohort design. The outcome measurement was the score on the Bayley Scales of Infant and Toddler Development at 15 months of age. Independent variables were gender, WAMI index, HOME score, weight-for-age z-score, length-for-age z-score, and head circumference-for-age z-score.

### Study setting

The setting is rural northern central Tanzania, in the Manyara region in villages surrounding Haydom (TZH). The population is mainly peasants living from mainly maize, beans farming and animal keeping. The village is of low economic status and without tarmac roads. Malnutrition is common among children under the age of five in the Manyara region, with a quarter of them underweight (weight-for-age below − 2 SD) [11] The study site is described more in detail elsewhere [12].

### The study population

The study’s catchment area was defined geographically and all pregnant women in their third trimester over a period of 2 years were asked to participate. Exclusion criteria were if the family had plans to move outside the area, if the mother was younger than 16 years of age, twin pregnancy, born underweight (< 1.5 kg), or if they already had a child enrolled in the study. Infants participated in repeated household visits; 262 infants were enrolled within 17 days of birth. For the present analysis, 137 children with Bayley scores at age 15 months (455 ± 15 days) were included in the analysis.

### Study instruments

The third edition of the Bayley Scales for Infant and Toddler Development was used to assess cognitive, language and motor development [13]. The test includes various questions, scenarios and tasks and takes approximately 45–60 min to complete. The test was administered by a trained person and conducted at 15 months of age. Details about translations and needed adaptations (e.g. replacing “foreign” items such as snow and vacuum cleaners) are described elsewhere [14].

Socioeconomic status was measured at the 6-month follow-up using a socioeconomic questionnaire. A WAMI index (scale from 0 to 1) [15], accounting for a household Water and sanitation type, various Assets, Maternal education and monthly Income is used in this analysis (Box 1).

The Home Observation for Measurement of the Environment (HOME) inventory, an instrument developed and validated by Caldwell and Bradley [16], was used to assess quality of the child´s home environment. The HOME inventory was also taken at 6 months of age (Box 2).

**Box 1:** Calculation of the Water/sanitation, Assets, Maternal education, and Income (WAMI) index (Psaki et al.)

	Description	Range
Water/sanitation	Using WHO definitions of access to improved water and improved sanitation, households with access to improved water or improved sanitation are assigned a score of 4 for each. Households without access to improved water or improved sanitation are assigned a score of 0 for each. These scores were summed	0–8
Assets	Eight priority assets were selected. For each asset, households were assigned a 1 if they have the asset and 0 if they do not have the asset. These scores were summed	0–8
Maternal education	Each child’s mother provided the number of years of schooling she had completed, ranging from 0 to 16 years. This number was divided by 2	0–8
Income	Monthly household income was converted to US dollars using the exchange rate from January 1, 2010. Income was divided into octiles using the following scores and cutoffs: 1(0–26), 2 (26.01–47), 3 (47.01–72), 4 (72.01–106), 5 (106.01–135), 6 (135.01–200), 7 (200.01–293), 8 (293 +)	0–8
TOTAL	Scores in water and sanitation, assets, mother´s education, and income were summed then divided by 32	0–1

**Box 2:** Description of calculation of the adjusted HOME Inventory for the Tanzanian site

HOME category	Description	Range
Emotional and verbal responsivity	Caregiver tells the child the name of some object or says the name of a person or object in a teaching style during the visit. (0–1) Caregiver’s speech is distinct, clear, and audible. (0–1) Caregiver initiates verbal exchanges with the observer—asks questions, makes spontaneous comments. (0–1) Caregiver expresses ideas freely and easily, and uses statements of appropriate length for conversation (i.e., gives more than brief answers). (0–1) Caregiver spontaneously praises child’s qualities or behavior at least twice during visit. (0–1) Caregiver shows some positive emotional response or praise to the child offered by the observer. (0–1) Caregiver smiles at the child or laughs with the child. (0–1)	0–7
Organization	When the primary caregiver is away, care is provided by one of three regular substitutes. (0–1) The child has a special place to keep his toys and “treasures.” (0–1) The child’s play area is relatively safe and free from hazards. (0–1) The stove is located in a relatively safe area. (0–1) The house is relatively light. (0–1) The house is relatively ventilated. (0–1) The house is relatively clean. (0–1) The house is relatively neat and orderly. (0–1)	0–8
Opportunities	The caregiver sings to the child everyday. (0–1) The family visits or receives visits from relatives at least once per month. (0–1) The family visits or receives visits from close friends at least once per month. (0–1)	0–3
Cleanliness of child	The child is relatively clean, with no offensive odor. (0–1) The child’s hair is relatively clean. (0–1) The child’s clothes are relatively clean. (0–1)	0–3

Nutritional status was assessed using anthropometric measurements. Weight at enrollment was examined and z-scores for length, weight and head circumference at enrollment and at 6 months of age were calculated using WHO child growth standards.

### Data collection and analysis

Data was collected by trained local field staff. Statistical analyses were conducted using SPSS Version 23. The Bayley assessments were all video recorded and evaluated locally, and 10% of the videos were sent off-site for another quality check performed by a trained Bayley administrator. These evaluations revealed that one out of four of the local Bayley examiners did not have the necessary quality in the assessment. All included assessments were analyzed for psychometric properties in order to check for scale reliability. Bayley assessment was done at 15 months, because at 12 and 18 months there were too many other assessments in this “MALED” study. Some data was missing, and some was tested too late, and one field assistant for Bayley assessment was excluded.

### Statistical analysis

Following the procedure described in length by Lyngmo et al. [17] the subscales of Bayley were revised as following. All items with zero variance were removed, thereafter all items with < 0.30 item-total correlation. This yielded a revised measurement consisting of four subscales: Cognitive (23 items, Cronbach alpha 0.85), Language (15 items, Cronbach alpha 0.89), Fine motor (12 items, Cronbach alpha 0.71), and Gross motor (22 items, Cronbach alpha 0.82). These four scales as well as the total score were used as the outcome measures. Using the same procedure with the HOME inventory psychometric properties were analyzed and adjusted in order to be more reliable in this setting. Items with no variance were excluded and correlations coefficients were calculated. All items with correlations coefficients less than 0.30 were excluded one by one until satisfactory correlations were obtained. As a result some subscales were omitted and we kept the following: seven items on emotional and verbal responsivity (Cronbach alpha 0.66), eight items on organization of physical and temporal environment (Cronbach alpha 0.72), three items on opportunities for variety in daily stimulation (Cronbach alpha 0.68), and three items on cleanliness of child (Cronbach alpha 0.83). Each item in both HOME and Bayley were scored as either 0 or 1, making the maximum score the same as the number of items for that scale.

Means and standard deviations were calculated for continuous data and proportions for categorical data. Variables were tested for normality. Potential associations between 15-month Bayley scores and selected determinants were analyzed by univariate linear regression analysis, with 5% significance level. The associations are presented as adjusted Beta regression coefficients.

A multivariate model was built for the cognitive scale of the Bayley, retaining potential explanatory variables where the *p* value in univariate regression was less than 0.1, and then running a forward stepwise linear regression. Gender and tribe were included in the multivariate model as a control factors. In our model male gender was 1 and “female” was 2, and for Iraq tribe was 1 and “others” was 2.

### Ethical issues

The study was approved by the Tanzanian National Institute for Medical Research and Ministry of Health and Social Welfare. Parents or legal guardians signed an informed consent form after the study’s objectives, procedures, risks, benefits, and confidentiality procedure were explained.

## Results

We screened 274 pregnant women. None of these declined and all were over 16 years. Three mothers were not able to give informed consent, seven children were not healthy and two pregnancies were twins, hence not eligible. There were 262 presumably healthy singleton children enrolled into the study, and we included the 135 children who had data for 15-month Bayley scores; 77 (56.2%) were girls (Table 1). Mean weight at enrollment (within 17 days after birth) was 3.36 kg (SD = 0.5) and 3.6% of the children had a bodyweight of 2500 grams or less at enrollment. Almost all of them (97%) started breastfeeding within 24 h. Apgar score was not available as most of the births were at home (54%).

**Table 1:** Baseline characteristics of 137 children in rural Tanzania

Variables	n	Mean	Standard deviation	%
Bodyweight at enrolment (0–17 days)	137	3.39 kg	0.49	
WAZ at enrolment (0–17 days)	137	− 0.13	0.91	
Gender	137			
Male	60			43.8
Female	77			56.2
Tribe				
Iraqw	103			85.8
Datog	10			8.3
Other	7			2.5
Missing	17			2.5
Nutritional status				0.8
LAZ at 6 months	137	− 1.24	1.05	
LAZ ≤ − 2 (stunted)	28			20.4
WAZ at 6 months	137	− 0.60	1.12	
WAZ ≤ − 2 (wasted)	14			10.2
WLZ at 6 months	137	0.32	1.20	
WLZ ≤ −2 (malnourished)	4			2.9
Socioeconomic status				
WAMI index	135^b^	0.21	0.12	
Sanitation^a^	135^b^	1.81	2.17	
Assets	135^b^	1.90	1.64	
Maternal education (years)	135^b^	5.20	2.78	
Income per month (TZH shilling)^a^	135^b^	42,861	58,698	
Mother’s age (years)	121^c^	29.31	6.61	
Mother’s number of pregnancies^a^	122^d^	4.70	2.81	
Home environment				
Emotional and verbal responsivity^a^	137	6.70	0.85	
Organization	137	3.04	2.18	
Opportunities^a^	137	2.77	0.58	
Cleanliness of child^a^	137	2.48	0.93	
Cognitive development at 15 months				
Bayley total score	137	43.15	8.55	
Bayley cognitive score	137	11.50	4.13	
Bayley language score	137	8.26	3.99	
Bayley fine motor score^a^	137	10.94	1.43	
Bayley gross motor score	137	12.45	2.45	

*LAZ* length-for-age z-score; *WAZ* weight-for-age z-score; *WLZ* weight-for-length z-score; *WAMI* index water, assets, maternal education, and income

^a^Not normally distributed

^b^Missing 2

^c^Missing 16

^d^Missing 15

Socioeconomic indicators showed that the mothers’ mean years of schooling were 5.2 (SD = 2.8). The mean family income per month was 42,860 Tanzanian shillings (20 USD). The mean age of their mothers was 29.3 years (SD = 6.6) and they had a mean number of pregnancies of 4.7 (SD = 2.8). The children’s mean 15-month Bayley scores are shown in Table 1.

In univariate regression analysis weight-for-age and weight-for-length z-scores at 6 months were significantly associated with 15-month Bayley gross motor score, but not with other 15-month Bayley scores (Table 2). Length-for-age z-scores at 6 months were not significantly associated with 15-month Bayley scores.

**Table 2:** Univariate linear regression analysis of determinants of Bayley scores at 15 months of age in 137 children in rural Tanzania

Determinants at 6 months	Bayley scores at 15 months									
	Cognitive score		Language score		Fine motor score		Gross motor score		Total score	
	Beta	p	Beta	p	Beta	p	Beta	p	Beta	p
Gender^a^	0.18	0.03	0.12	0.15	0.11	0.21	0.05	0.58	0.18	0.04
Tribe^b^	0.04	0.69	0.14	0.12	0.08	0.36	0.02	0.84	0.10	0.26
Nutritional status										
Length-for-age z-score	0.05	0.56	0.05	0.56	0.11	0.22	0.03	0.77	0.06	0.50
Weight-for-age z-score	0.01	0.95	0.05	0.60	0.01	0.91	0.18	0.03	− 0.03	0.73
Weight-for-length z-score	0.02	0.80	0.03	0.77	0.09	0.28	0.19	0.03	− 0.07	0.42
Socioeconomic status										
WAMI index	0.17	0.05	0.02	0.83	0.04	0.67	0.06	0.46	0.12	0.18
Sanitation	0.01	0.92	0.07	0.40	0.00	0.99	0.10	0.26	0.06	0.51
Assets	0.21	0.01	0.03	0.76	0.06	0.49	0.000	1.00	0.10	0.25
Maternal education	0.08	0.35	0.01	0.92	0.03	0.75	0.10	0.27	0.08	0.39
Monthly income	0.20	0.02	0.03	0.72	0.03	0.71	0.05	0.60	0.08	0.39
Home environment										
Emotional and verbal responsivity	0.05	0.60	0.09	0.28	0.06	0.50	0.05	0.53	0.06	0.49
Organization	0.12	0.17	0.05	0.58	0.09	0.28	0.07	0.42	0.07	0.42
Opportunities	0.04	0.67	0.08	0.38	0.05	0.55	0.07	0.39	− 0.08	0.34
Cleanliness of child	0.15	0.08	0.15	0.09	0.05	0.60	0.16	0.07	− 0.19	0.02

Beta is the regression co-efficient

^a^In the regression model male = 1 and female = 2

^b^In the regression model Iraqw = 1 and others = 2

The cleanliness of child at 6 months from the HOME inventory scale was significantly associated with 15-month Bayley total score, but not with any of the other 15-month Bayley scores. The cleanliness variable was not normally distributed as it was strongly skewed to the right, meaning higher level of cleanliness. Other scores from the HOME inventory were not significantly associated with 15-month Bayley scores.

The WAMI index, assets component, and monthly income were significantly associated with 15-month Bayley cognitive score, but not with language, motor, nor total 15-month Bayley scores. Other socioeconomic variables in the WAMI index were not significantly associated with 15-month Bayley scores.

Multivariate regression analysis of cognitive Bayley, showed statistically significant associations with gender and socioeconomic status, with higher Bayley cognitive scores for girls, and higher Bayley cognitive scores in families with more assets and income from the WAMI index. Adjusted R-squared in this model was 8% (Table 3).

**Table 3:** Multivariate linear regression analysis of determinants of Bayley cognitive score at 15 months of age in 137 children in rural Tanzania

Determinants	Bayley cognitive score at 15 months	
	Beta	p
Gender^a^	− 0.20	0.03
Tribe^b^	0.06	0.51
Assets	0.24	0.01

Adjusted r-squared 7.6%

^a^In the regression model male = 1 and female = 2

^b^In the regression model Iraqw = 1, others = 2

## Discussion

Our analysis aiming at identifying factors thought to influence early child development shows that socioeconomic factors were associated with cognitive development, and nutritional status was associated with gross motor development.

Our analysis shows that the strongest factor correlated with child cognitive development at 15 months of age is the socioeconomic status of the household. The robust association between socioeconomic status and child development is well known [1, 18–20]. The Lancet series on children’s development in low-income countries concluded that low socioeconomic status was a determinant of poor development [1]. This effect can be seen before birth and continue into adulthood [21]. Socioeconomic factors may affect childhood brain development through a variety of mechanisms, such as prenatal factors, parental care, cognitive stimulation, toxin exposure, nutrition, and stress [22, 23]. A study from Greece showed that maternal education, which is a component of the WAMI index, and which is often itself associated with socioeconomic status, was positively correlated with child development [24]. A study on the effects of socioeconomic status on neurocognitive systems showed that the association is most pronounced with language and memory [25]. One study using magnetic resonance imaging of the brain found that lower family socioeconomic status was associated with smaller volumes of gray matter in the brain [26], and consequently environmental factors may have detrimental effects on brain structure and function.

Stunting and wasting are indicators that reflect nutritional status. Stunting (length-for-age z-score (LAZ) < -2) is an indicator of chronic malnutrition and wasting (weight-for-length z-score (WLZ) < -2) is an indicator of acute malnutrition [27]. Our analysis suggests that both stunting and wasting at 6 months of age are associated with poorer motor development at 15 months of age. A study from Pemba, Tanzania showed that higher LAZ scores were significantly associated with better motor and language development [28]. Many other studies from Africa to Asia show that better nutrition is positively correlated with child development [27, 29–32].

Lower socioeconomic status is associated with lower nutritional status, poor sanitary and hygiene conditions, which in turn is associated with higher rates of infections and stunting in children. All of these factors intertwine and contribute to a child’s development [33, 34]. This demonstrates the interaction between socioeconomic status and nutritional status, and it also highlights the complex interaction between environmental factors on child development.

Cleanliness of one’s child was associated with total Bayley score. We do not have any clear explanation for this; there could be some information bias as the pregnant women learn about cleanliness in schools and at mother-and-child clinics and may respond correspondingly, but we also speculate that in poor households like these the cleanliness of the child is the last thing to give up.

In this study there was a significant association between girls’ and cognitive Bayley score, also reflected in the total score. We do not have any good explanation for this. Some information bias is possible, as items used for measuring these Bayley scores may have been more familiar to female infants and hence influencing the score. The difference between tribes is also difficult to explain; some have different culture and habits like hygiene and hence the items used for Bayley scores may turn out somehow biased. The Iraqw tribe may have more of their extended family close to home and hence potentially more support from extended family.

We observed a better nutritional status was associated with better gross motor Bayley score, but not fine motor Bayley score. We do not have any explanation of this but speculate that gross motor score is more influenced by undernutrition because of less physical strength, whereas fine motor score is less dependent on this.

### Strengths and limitations

This study has several strengths. It is part of a large multi-centre research project studying determinants of child development, and with a strong research group coordinating the study sites [10]. The study tools used were acknowledged and tested, and data collection was done under close continuous supervision [14].

Some limitations of this study should be noted. First, in this observational study we cannot prove any causal relationships, only statistical associations. Second, some determinants of child development were not examined; for example, genetic factors, neighborhood processes, conflict and violence [23]. Third, the Bayley and the HOME inventory were modified in order to be more applicable in the local rural Tanzanian setting, and hence their validity was not yet evidence-based in all aspects. Fourth, unfortunately, as explained in Methods, a substantial proportion (52%) of the enrolled participants did not have their Bayley test taken at 15 months of age and were excluded from analysis, limiting the sample size and statistical power. As this also posed a risk of selection bias we compared the excluded and included participants by sex (p = 0.27), initial bodyweight (p = 0.57), initial z-score (p = 0.52) and 12 m WAMI (p = 0.27), and concluded that the excluded participants were very probably comparable to those included. The minimal sex difference among the participants included and those excluded causes slight concern as this sex is a weak risk factor, and could slightly affect the associations studied. Finally, in this area all households were poor to some degree and the variation in SES was not large; consequently this results in low statistical power in analysis of these associations.

## Conclusions and implications

We conclude that lower socioeconomic status is associated with poor cognitive development in children and malnutrition is associated with reduced gross motor development. This information should encourage authorities and other stakeholders to invest in improved welfare and nutrition programmes for children from early infancy.

## Abbreviations

MAL-ED: The Interactions of Malnutrition & Enteric Infections: Consequences for Child Health and Development
Bayley: Bayley Scales of Infant Development
TZH: Tanzanian site
WAMI: Water and sanitation type, various Assets, Maternal education and monthly Income
HOME: Home Observation for Measurement of the Environment inventory
LAZ: length-for-age z-scores
WAZ: weight-for-age z-scores
WLZ: weight-for-length z-score
